# Fractures of the Humeral Shaft with Primary Radial Nerve Palsy: Do Injury Mechanism, Fracture Type, or Treatment Influence Nerve Recovery?

**DOI:** 10.3390/jcm8111969

**Published:** 2019-11-14

**Authors:** Roman C. Ostermann, Nikolaus W. Lang, Julian Joestl, Leo Pauzenberger, Thomas M. Tiefenboeck, Patrick Platzer

**Affiliations:** 1 St. Vincent Shoulder & Sports Clinic, 2nd Orthopedic Department, Hospital of the Sacred Heart of Jesus, 1030 Vienna, Austria; leo.pauzenberger@kh-herzjesu.at; 2 Department of Orthopedics and Trauma Surgery, Medical University of Vienna, A–1090 Vienna, Austria; nikolaus.lang@meduniwien.ac.at (N.W.L.); julian.joestl@meduniwien.ac.at (J.J.); thomas.tiefenboeck@meduniwien.ac.at (T.M.T.); patrick.platzer@meduniwien.ac.at (P.P.); 3 Department of Trauma Surgery, University Hospital of St. Poelten, Karl Landsteiner University of Health Sciences, 3500 Krems an der Donau, Austria

**Keywords:** radial nerve palsy, humeral shaft fracture, ORIF, intramedullary nailing, functional outcome

## Abstract

Adult humeral shaft fractures are associated with primary radial nerve palsy in up to 18% of cases. The purpose of this study was to assess the influence of injury mechanism, fracture type, and treatment on nerve recovery in patients with humeral shaft fractures and primary nerve palsy. Data of fifty patients (age—43.5 ± 21.3; female: male—1:1.8) with humeral shaft fractures and concomitant grade I–II primary radial nerve palsy, who underwent either open reduction and internal fixation (ORIF) or intramedullary nailing at an academic level I trauma center between 1994 and 2013, were evaluated. Factors potentially influencing the time to onset of recovery or full nerve recovery (injury mechanism, fracture type, fracture location and treatment) were analyzed in detail. Thirty patients were treated with ORIF and twenty patients with closed unreamed intramedullary nailing of the humeral shaft, respectively. The mean time to onset of recovery was 10.5 ± 3.4 weeks (2–17 weeks). Twenty-six (52%) patients reported significant clinical improvement within the first 12 weeks. Mean time to full recovery was 26.8 ± 8.9 weeks (4–52 weeks). Twenty-five (50%) patients regained full manual strength within the first six months following the injury. Forty-nine (98%) patients regained full manual strength within the first 52 weeks. Trauma mechanism, fracture type, fracture location, and treatment modality did not influence the time to onset of nerve recovery or time to full recovery following humeral shaft fractures with grade I–II primary radial nerve palsy.

## 1. Introduction

Adult fractures of the humeral shaft account for approximately 3% of all fractures [1]. Due to the anatomy of the radial nerve and the entrapment between fragments in spiral fractures of the humerus, these injuries are associated with primary radial nerve palsy in up to 18% [2,3]. Both open reduction and internal fixation (ORIF) with plate osteosynthesis, and intramedullary nailing, are well-established treatment methods [1,4]. The choice of operative treatment for a humeral shaft fracture depends on multiple factors: (1) fracture indications; (2) associated injuries; and (3) patient indications. Certain advantages of intramedullary nailing, such as minimal invasive insertion techniques, intact periosteal blood supply, less stress shielding at the fracture site, and shorter average operation times accompanied with less blood loss, have been mentioned [4,5,6,7]. However, the main disadvantages of intramedullary nailing are less anatomic fracture reduction and potential subacromial impingement syndrome with consecutive restriction of shoulder movement [4,5]. ORIF, on the one hand, allows for direct visualization of the fracture site and the radial nerve, facilitating possible dissection of an entrapped radial nerve as well as anatomic reduction and therefore reducing the risk of non-union [8]. However, due to the open surgical approach to the humerus, ORIF potentially increases the risk of iatrogenic radial nerve damage during soft tissue preparation, leads to longer operation time and greater blood loss [4,5,6,7]. 

In the case of primary radial nerve palsy, the decision of whether or not an additional early nerve exploration is indicated has to be made [3,9]. Due to high rates of spontaneous recovery, reserved surgical nerve exploration in cases of primary radial nerve palsy has been postulated [9]. Although some national guidelines aim to give clear treatment recommendations [10], the management of humeral shaft fractures with primary radial nerve palsy, as well as the best method of fracture fixation, still remains challenging and a worldwide consensus regarding the optimal type of treatment is missing [5,6,11,12].

Therefore, the purpose of this study was to assess the influence of injury mechanism, fracture type, and type of surgical treatment, on time to onset of nerve recovery and time to full nerve recovery in patients with humeral shaft fractures and concomitant primary radial nerve palsy.

## 2. Material and Methods

The study was approved by the local ethics committee and was performed in accordance with the Declaration of Helsinki. Written informed consent was obtained from all patients before enrolment in this study. This study was retrospectively registered at clinicaltrials.gov with the number NCT03948542.

### 2.1. Study Design and Patient Recruitment

**Figure 1:**
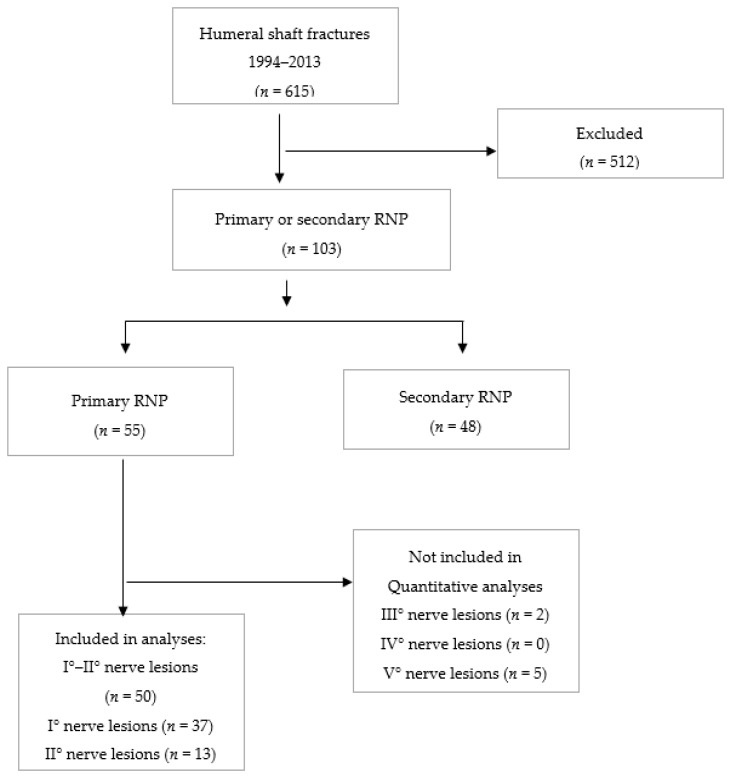
Detailed overview of study inclusion and exclusion criteria.

An analysis of prospectively-collected data for all patients treated with humeral shaft fracture and primary radial nerve palsy at an academic Level 1 trauma center was performed. Between 1994 and 2013, a total of 615 patients with traumatic humeral shaft fractures were treated at the department. The dataset was examined for completeness and accuracy. Patients with an incomplete dataset, those with a pathologic or periprosthetic fracture, patients with non-surgical treatment, or who were younger than 18 years of age, were excluded from this series. According to these criteria, a total of 50 patients with a humeral shaft fracture and primary radial nerve palsy underwent surgical treatment (Figure 1). Patient demographics are summarized in Table 1.

### 2.2. Diagnosis and Surgical Treatment

All humeral shaft fractures were diagnosed by two plane standard X-ray images and classified according to the AO (Arbeitsgemeinschaft Osteosynthese) classification system [13]. Additionally, the location of the fracture along the shaft (proximal, mid-shaft, distal) was noted. Severity of soft tissue damage was classified according to the Gustilo–Anderson classification [14].

Fracture fixation was performed either by open reduction and internal fixation using a 4.5 mm, limited-contact dynamic compression plate through an anterolateral approach, or locking unreamed intramedullary antegrade or retrograde nailing (IN) through minimally-invasive standard approaches. The surgeries were performed by a senior surgeon experienced with treating this kind of fracture or under supervision. The grade of nerve damage was determined according to the Sunderland’s classification with the help of nerve conduction surveys [15]. All patients underwent a neurologic rehab protocol under guidance of a physiotherapist following discharge from hospital.

### 2.3. Outcome Assessment

Clinical and radiographic examination was routinely performed 3, 6, and 12 months after the trauma. Radiographs in two planes were taken to determine fracture healing and position of the fragments or any loosening of the implants. Nerve conduction studies (NCVs) were performed routinely at two weeks following onset of radial nerve palsy, and after four months in case of delayed recovery. Functional assessment was routinely performed at all follow-up visits including a clinical evaluation and muscle strength (M0–M5) with a manual muscle test that was graded according to Daniels and Worthingham [16]. Onset of recovery was defined as clinically recognizable improvement from initially-documented muscle strength evaluated by manual testing. Time to full recovery was defined as the time from surgery to restoration of full muscle strength (M5) on manual muscle testing, or restoration to near-full muscle strength (M4) if there was no improvement noted up to the latest documented follow-up.

### 2.4. Statistical Analysis

Quantitative data were compared between the two groups using a factorial ANOVA and a Mann Whitney U test. Qualitative data were compared using the χ^2^ or Fisher’s exact test. Multiple regression analysis with a 95% confidence interval was used to examine the independent associations of injury-related factors (traumatic mechanism, fracture type, and treatment). Age and sex were excluded from the multiple regression analysis due to multicollinearity. Statistical significance was set at the conventional *p* < 0.05.

## 3. Results

The mean time to onset of recovery was 10.5 ± 3.4 weeks, ranging from 2 to 17 weeks. Twenty-six (52%) patients reported significant clinical improvement within the first 12 weeks.

Mean time to full recovery was 26.8 ± 8.9 weeks, ranging from 4 to 52 weeks. Twenty-five (50%) patients regained full manual strength within the first six months following the injury. Forty-nine (98%) patients regained full (M5) manual strength within the first 52 weeks. One patient had regained almost full manual strength (M4) at 52 weeks, with no significant changes thereafter. 

High-energy trauma was significantly higher in male patients (*p* < 0.001) and patients under 40 years of age (*p* < 0.001). Surgical management was performed with an average delay of 1.8 days, ranging from 0 to 6 days. 

Type A fractures were more commonly treated with IN (*p* < 0.001), whereas Type B and Type C fractures were significantly more often treated with ORIF (*p* < 0.001). Among the patients treated with IN, 8 patients underwent additional surgical exploration of the radial nerve by a small incision directly over the fracture site during the same surgery, whereas in 12 patients, no further surgical investigation of the nerve was considered necessary. The decision to carry out an additional exploration of the radial nerve was based on surgeon preference.

Analysis of fracture locations along the shaft revealed a mid-shaft fracture location with a distal fracture extension in 17 cases, and a distal shaft fracture location in the remaining 33 cases. Out of the mid-shaft fractures with a distal fracture extension, 10 were treated with IN and 7 with ORIF, whereas 23 of the distal shaft fractures were treated with ORIF and only 10 with IN (*p* < 0.071).

There were three open fractures in the study group, including two grade I and two grade II injuries according to the Gustilo–Anderson Classification. Patients with open fractures did not show statistically longer time to onset of recovery or time to full recovery than patients with closed fractures.

There was no difference in time to onset of nerve recovery or time to full nerve recovery between grade I and II radial nerve lesions. There was no significant difference in time to onset of nerve recovery or time to full recovery between high- and low-energy traumas, between fracture types and fracture locations along the shaft or type of treatment (Table 2). Furthermore, multiple regression analysis did not show any significant influence of these factors on time to onset of recovery (Table 3) or time to full recovery (Table 4).

## 4. Discussion

Summarizing our results, they revealed type A fractures to be treated significantly more commonly with IN compared to Type B and C fractures, which were treated with ORIF more often. High-energy trauma as a cause was significantly more often found in male patients and patients under the age of 40 years. However, there was no difference in time to onset of nerve recovery or time to full nerve recovery between grade I and II radial nerve lesions. There was also no significant difference in time to onset of nerve recovery or time to full recovery between high- and low-energy traumas, between fracture types, or the type of treatment.

The radial nerve is the nerve most frequently injured with fractures of the humeral shaft due to its spiral course across the back of the mid-shaft of the bone [2,3]. Because the nerve is usually only bruised or stretched, function can be expected to return spontaneously [2,9]. 

If radial nerve palsy occurs with an open fracture of the humeral shaft, the nerve should be explored at the time of debridement of the wound [3,9]. However, in case of a closed humeral shaft fracture with primary radial nerve palsy, recent literature reveals no worldwide consensus regarding optimal surgical treatment. Treatment algorithms regarding fracture fixation and therapy of radial nerve palsy vary between different trauma centers [4,11]. However, national guidelines exist that aim to provide optimal treatment recommendations regarding humeral shaft fractures and primary radial nerve palsy [10]. Both ORIF with dynamic compression plates and intramedullary nailing are considered safe and reliable procedures [1,5,6,12]. In a prospective study, Chapman et al. [17] could not find significant statistical differences between the two treatment options, showing that they are both equal in terms of fracture healing. Recent studies reported similar rates of nerve recovery in patients with primary radial nerve palsy among patients treated with ORIF (85%) or unreamed intramedullary nails (93%) [7,8]. In support of those findings, we observed no differences regarding functional outcome and recovery in patients undergoing ORIF or intramedullary nailing in our comparative series. 

It has been stated that in cases of acute primary radial nerve palsy in traumatic humeral shaft fractures, open reduction facilitates fracture treatment and simultaneous exploration of the radial nerve to determine the extent and the type of lesion [1,11,18]. However, in the case of low-energy trauma, radial nerve palsy is often caused by simple nerve contusion or stretching with the nerve being found usually macroscopically intact [9,19,20]. Thus, watchful waiting seems reasonable in patients sustaining low-energy trauma. The mostly higher age of these patients and the higher incidence of comorbidities might further support the avoidance of surgical exploration in those cases [1,14]. 

It has been stated that time to recovery of radial nerve palsy depends on factors like trauma mechanism, fracture type, and surgical management [7,8,9,12,21,22,23]. Shao et al. [11] published in their review that for the rate of spontaneous nerve recovery of primary radial palsy with an average time of onset of 7.3 weeks (2 weeks to 6.6 months), the time of full recovery was reported as 6.1 months (3.4–12 months) [11]. In contrast, Venouziou et al. reported in their series that in patients with low-energy trauma, the initial time to recovery was 3.2 weeks (1–8 weeks) and the average time to full recovery was 12 weeks (3–22 weeks), whereas in patients following high-energy trauma, the time of initial recovery was 14 weeks (7–23 weeks) and the mean time to full recovery was 26 weeks (11–35 weeks) [20]. However, this finding cannot be supported with our results, since we did not find a significant influence of the trauma mechanism on time to onset or time to full recovery. On the other hand the mean onset of spontaneous recovery was at 10.5 weeks and full recovery was achieved at about 26.8 weeks following surgery in our case series, which seems to be consistent with findings described in the current literature [7,8,9,11]. 

It was to our surprise that, in contrast to existing literature, we observed no significant influence of fracture type on recovery of radial nerve palsy [9,12,15,16,20,21]. The reasons for these differences remain unclear at this time with further studies being necessary to investigate possible influencing factors beyond what has been previously investigated in the literature. Nevertheless, one possible explanation for our finding could be the fact that the AO classification might simply not be sufficient enough to detect for influences on nerve recovery. A simple type A fracture in a region where the nerve is in close proximity to the bone might represent a greater risk for nerve damage than a complex type C fracture further away. Hence, the localization of the fracture might be of more importance regarding possible nerve damage, than the complexity of the fracture. We therefore additionally analyzed whether the localization of the shaft fracture had an influence on the time to onset and time to full recovery of the radial nerve palsy, but again, were unable to detect one.

There are several limitations to this study. We retrospectively evaluated a small sample size, analyzing an inhomogeneous patient population, using data collected by multiple examiners over two decades. In combination, this made it impossible to report clinical outcome beyond the measure of manual muscle strength, which was uniformly available for all patients. The chosen treatment modality was highly dependent on the type of fracture, which represents a selection bias.

Nonetheless, the current study has several strengths. To the best of our knowledge, it is the first series to focus on the influence of the injury mechanism, fracture type, fracture location, and type of treatment on recovery from primary radial nerve palsy associated with traumatic humeral shaft fractures. Furthermore, although our sample size was small, we present a relatively high number of evaluated cases compared to the existing literature.

In conclusion, the type of surgical treatment, ORIF or intramedullary nailing, revealed no significant influence either on time to onset of nerve recovery, or time to total recovery, and no significant improvement in humeral shaft fractures with primary radial nerve palsy.

## Figures and Tables

**Table 1:** Patient demographics.

Patients	*n* = 50
Age	43.5 ± 21.3 years
**Sex**	
Female	18 (36%)
Male	32 (64%)
**Trauma mechanism**	
High-energy	29 (58%)
Low-energy	21 (42%)
**Fracture type**	
A1	*n* = 17 (34%)
A2	*n* = 5 (10%)
A3	*n* = 7 (14%)
B1	*n* = 14 (28%)
B2	*n* = 2 (4%)
B3	*n* = 3 (6%)
C1	*n* = 2 (4%)
**Treatment**	
Intramedullary nailing	*n* = 20 (40%)
ORIF	*n* = 30 (60%)

Values are given as frequency and proportion or mean ± SD wherever appropriate. ORIF—open reduction and internal fixation.

**Table 2:** Comparisons of time to onset and time to full recovery.

	Injury Mechanism		*p*	Fracture Type							p *	Treatment		*p*	Fracture Location		*p*
	Low-Energy	High-Energy		A1	A2	A3	IN	IN	B3	C1		IN	ORIF		Mid-Shaft	Distal-Shaft	
**Time to onset of recovery** **(weeks)**	10.7 ± 3.1	10.3 ± 3.5	0.680	10.7 ±2.9	11.4 ± 4.0	11.4 ± 4.0	10.7 ± 3.5	10.7 ± 3.5	12.3 ± 2.9	10.5 ± 0.7	0.552	10.7 ± 3.5	10.3 ± 3.3	0.774	10.7 ± 3.4	10.4 ± 3.4	0.737
**Time to full recovery** **(weeks)**	28.1 ± 10.1	25.8 ± 8.0	0.387	26.2 ±8.3	25.0 ± 4.7	30.3 ± 9.0	25.4 ± 7.8	25.4 ± 7.8	26.0 ± 4.4	35.5 ± 2.1	0.728	25.4 ± 7.8	27.8 ± 9.6	0.353	27.0 ± 9.3	26.7 ± 8.9	0.911

Values are given as mean ± SD in weeks. * indicates significance upon one-way analysis of variance, the remaining levels of significance were obtained using an independent *t*-test. ORIF—open reduction internal fixation; IN—intramedullary nailing. Fracture type: A1, A2, A3, B1, B2, B3, C1

**Table 3:** Multiple linear regression analysis for factors potentially influencing time to onset of recovery.

	Β *	t **	p **	95% Confidence Interval *
**Trauma mechanism**	−0.312	−0.308	0.759	−2.352 to 1.728
**Fracture type**	−0.139	−0.464	0.645	−0.742 to 0.464
**Treatment**	−0.111	−0.043	0.966	−2.256 to 2.163
**Fracture location**	−0.248	−0.228	0.821	−2.436 to 1.941
***R*^2^ = 0.011**				

* One unit represents one-week difference between the tested factorial values. ** *t*, *p*: *t*-statistics and according *p*-value (two-tailed), an alpha level <0.05 was considered statistically significant. Trauma mechanism: low-energy, high-energy; fracture type: A1, A2, A3, B1, B2, B3, C1; treatment: intramedullary nailing, open reduction internal fixation; fracture location: mid-shaft, distal shaft.

**Table 4:** Multiple linear regression analysis for factors potentially influencing time to full recovery.

	Β *	t **	p **	95% Confidence Interval *
**Trauma mechanism**	−2.454	−0.928	0.358	−7.777 to 2.869
**Fracture type**	0.322	0.412	0.682	−1.251 to 1.895
**Treatment**	2.431	0.849	0.400	−3.335 to 8.196
**Fracture location**	−0.941	−0.332	0.741	−6.652 to 4.769
***R*^2^ = 0.041**				

* One unit represents a one-week difference between the tested factorial values. ** *t*, *p*: t-statistics and according p-value (two-tailed), an alpha level <0.05 was considered statistically significant. Trauma mechanism: low-energy, high-energy; fracture type: A1, A2, A3, B1, B2, B3, C1; treatment: intramedullary nailing, open reduction internal fixation; fracture location: mid-shaft, distal shaft.
